# Supplementary material for: WordCommentsAnalyzer: A windows software tool for qualitative research
Source: F1000Res. 2018 Sep 4;7:536. Originally published 2018 May 3. [Version 2] doi: 10.12688/f1000research.14819.2 (PMC6137411; doi:10.12688/f1000research.14819.2)
Supplement: Supplementary file 3 [file f1000research-7-17614-s0002.tgz › b2a79496-4ada-47c4-9682-a99ce867836d.zip!/Eight Iranian Health Journals/Journal of Research in Rehabilitation Sciences _50.docx]

© Journal of Research in Rehabilitation Sciences . This work is licensed under CC BY-NC 4.0 (https://creativecommons.org/licenses/by-nc/4.0/).

Volume & Issue: دوره 4، شماره 2: 1387

Title: بررسی تئوریک راه‌های ممکن برای حل مسأله نامعینی در ناحیه کمری توسط سیستم اعصاب مرکزی

http://jrrs.mui.ac.ir/index.php/jrrs/article/view/40

سيستم حرکتي انسان جهت انجام وظايف حرکتي مختلف با روش⁭های گوناگون و قابل انعطافي سازمان⁭دهي شده است. اين موضوع در اصل توسط راه⁭هايي انجام مي⁭شود که سيستم اعصاب مرکزی (Central nervous system) براي حل مسأله نامعينی (Redundancy) در سيستم اسکلتي- عضلاني به کار مي⁭گيرد. در واقع، نامعيني سينتيکي در سيستم اسکلتي- عضلاني انسان يک خصوصيت برجسته است که به وسيله آن سيستم اعصاب مرکزي مي⁭تواند به اهداف بسياري دست پيدا کند. در اين مطالعه ضمن ارایه تعاريف و بيان نقش مانيفولد کنترل نشده (Uncontrolled manifold) براي سينماتيک حرکت، مفهوم نامعيني سينتيکي با روابط رياضي بيان مي⁭شود. فضاي پوچ (Null space) سيستم با نامعيني سينتيکي، هنگامي که گشتاور و يا سختی (Stiffness) مشخصي نياز است، استخراج و روي آن بحث مي⁭شود. در این مقاله با استفاده از يک مدل بيومکانيکي 3 عضله⁭ای، روابط رياضي براي يک مدل صفحه⁭اي ساده استخراج شده است.

Title: تأثیر بیومکانیکی چسب زدن بر روی استخوان پاتلا (کشکک) در افراد مبتلا به سندرم درد مفصل پاتلوفمورال (PFPS)

http://jrrs.mui.ac.ir/index.php/jrrs/article/view/41

مقدمه: سندرم درد مفصل پاتلوفمورال (PFPS (يكي از شايعترين اختلالات اسكلتي- عضلاني ناحيه زانو در بالغين به شمار ميرود. چسب زني ناحيه پاتلا (taping Patellar ،(روش درماني مؤثر جهت تسكين علايم در طيف وسيعي از اين دسته از بيماران ميباشد. اگرچه كاهش علايم دردناك ممكن است مربوط به تغيير يافتن نيروهاي وارد بر مفصل پاتلوفمورال متعاقب استفاده از چسب باشد، با اين وجود مكانيسم كاهش علايم به درستي مشخص نشده است. مواد و روش‌ها: جهت تعيين اثرات چسب زني پاتلا بر بيومكانيك مفصل پاتلوفمورال بيماران مبتلا به PFPS ،مطالعه‌اي از نوع مورد- شاهدي طراحي گرديد. با استفاده از يك سيستم آناليز كننده حركت و يك دستگاه سكوي ثبت نيرو، نيروي واكنشي مفصل پاتلوفمورال (PFJRF (مربوط به 18 فرد مبتلا به PFPS و تعداد مشابهي از افراد سالم گروه شاهد، مورد ارزيابي قرار گرفت. اين نيروها از زانوي آسيب ديده افراد مبتلا به PFPS ،قبل از اعمال چسب، در حين اتصال چسب به ناحيه زانو و در نهايت پس از برداشته شدن آن در خلال مرحله انقباض واگراي (Eccentric (فعاليت حركتي خميدگي يك طرفه زانو (squatting-leg Single (به دست آمد. ارزيابي مشابهي از همين نيروها، از زانوي سمت سالم بيماران و همچنين هر دو زانوي افراد گروه شاهد در خلال فعاليت حركتي مشابه انجام پذيرفت . يافته‌ها: ميانگين عددي PFJRF پس از يك دوره اعمال چسب بر روي استخوان پاتلا (1720 نيوتن، 303 = SD ،(كاهش معنيداري نسبت به حالت قبل از استفاده از چسب (2025 نيوتن، 347 = SD (پيدا كرد ( 0/05 < P .(مقادير عددي PFJRF مربوط به زانوي آسيب ديده بيماران مبتلا به PFPS در حالت قبل از استفاده از چسب ناحيه پاتلا و همچنين افراد سالم گروه شاهد (1922 نيوتن، 398 = SD (تفاوت معنيداري را نشان نميداد .(P > 05/0 ) بحث: كاسته شدن از نيروهاي وارد بر مفصل پاتلوفمورال در حين استفاده از چسب ناحيه پاتلا كه همزمان با كاهش محسوس درد در اين مفصل به وقوع پيوست، ممكن است دليل قانع كنندهاي براي مكانيسم كاهش درد، پس از استفاده از اين روش درماني در مبتلايان به PFPS باشد

Title: بررسي علل درد مزمن قدام زانو در نوجوانان ایرانی

http://jrrs.mui.ac.ir/index.php/jrrs/article/view/42

<!-- /* Font Definitions */ @font-face {font-family:"Cambria Math"; panose-1:2 4 5 3 5 4 6 3 2 4; mso-font-charset:1; mso-generic-font-family:roman; mso-font-format:other; mso-font-pitch:variable; mso-font-signature:0 0 0 0 0 0;} @font-face {font-family:"B Zar"; panose-1:0 0 4 0 0 0 0 0 0 0; mso-font-charset:178; mso-generic-font-family:auto; mso-font-pitch:variable; mso-font-signature:8193 -2147483648 8 0 64 0;} /* Style Definitions */ p.MsoNormal, li.MsoNormal, div.MsoNormal {mso-style-unhide:no; mso-style-qformat:yes; mso-style-parent:""; margin:0cm; margin-bottom:.0001pt; text-align:right; mso-pagination:widow-orphan; direction:rtl; unicode-bidi:embed; font-size:12.0pt; font-family:"Times New Roman","serif"; mso-fareast-font-family:"Times New Roman"; mso-bidi-language:AR-SA;} p.abst, li.abst, div.abst {mso-style-name:abst; mso-style-unhide:no; margin-top:3.0pt; margin-right:0cm; margin-bottom:0cm; margin-left:0cm; margin-bottom:.0001pt; text-align:justify; mso-pagination:widow-orphan; direction:rtl; unicode-bidi:embed; font-size:10.0pt; mso-bidi-font-size:11.0pt; font-family:"Times New Roman","serif"; mso-fareast-font-family:"Times New Roman"; mso-bidi-font-family:"B Zar";} .MsoChpDefault {mso-style-type:export-only; mso-default-props:yes; font-size:10.0pt; mso-ansi-font-size:10.0pt; mso-bidi-font-size:10.0pt;} @page Section1 {size:612.0pt 792.0pt; margin:72.0pt 72.0pt 72.0pt 72.0pt; mso-header-margin:36.0pt; mso-footer-margin:36.0pt; mso-paper-source:0;} div.Section1 {page:Section1;} --> مقدمه: درد مزمن قدام زانو در نوجوانان، یکی از شايع‌ترین شكايات به خصوص در جامعه ما مي‌باشد و این بیماران درصد بالایی از مراجعين ارتوپدي را تشكيل مي‌دهند. درد قدام زانو در اين سنين، از يك طرف مي‌تواند روي روند تحصيلي تأثير منفي گذارد و از طرف ديگر مي‌تواند فعاليت‌هاي فيزيكي و ورزشي را، كه جز نيازهاي ضروري اين محدوده سني است، دچار مخاطره نمايد. علل درد مزمن زانو در نوجوانان بر خلاف بالغين بسيار متنوع مي‌باشد كه بعضي از آن‌ها به راحتي قابل درمان است و بعضي نياز به روش‌هاي تشخيصي و درماني پيچيده‌تر و پر هزينه‌تري دارند. بررسي و تشحیص به موقع ضایعه مي‌تواند منجر به درمان سريع‌تر و مؤثرتر آن گردد. هدف از اين مطالعه، بررسي علل درد مزمن جلوي زانو در نوجوانان كشور ما بود. مواد و روش‌ها: در اين مطالعه مقطعی از بین بیماران مراجعه کننده به مراکز آموزشی درمانی شهر اصفهان، 198 نوجوان بين 18-8 سال، كه بيش از 3-2 هفته درد جلوي زانو داشتند و با روش‌هاي درماني دارويي بهبود نيافته بودند، به مدت دو سال از آذرماه 1384 تا آذرماه 1386 بررسي شدند و يافته‌‌هاي شرح حال، معاينه باليني و اقدامات آزمايشگاهي و پاراكلينيك مورد نياز در يك پرسش‌نامه ثبت گردید؛ سپس داده‌ها مورد تحليل و بررسي قرار گرفت. يافته‌ها: درد مزمن زانو در پسران (1/53 درصد) شايع‌تر از دختران 9/46 درصد بود. شايع‌ترين علل درد مزمن جلوي زانو در نوجوانان به ترتيب شيوع عبارت از ديس پلازي پاتلوفمورال (Patella femoral dysplasia)، امتداد نامناسب اندام تحتاني (Malalignment)، ضايعات ليگاماني و منيسك‌هاي زانو، بيماري اسگود- شلاتر (Osgood-schlatter disease)، آرتريت روماتویید جوانان و استئوكندريت ديسكانت (Osteochondritis dissecans) بود. بحث: در اين مطالعه مشابه مطالعات ديگر، عللي همچون ديس پلازي پاتلوفمورال و امتداد نامناسب اندام بيشترين علل درد مزمن جلوي زانو در نوجوانان بود. عللي مانند امتداد نامناسب اندام و آرتريت روماتویید جوانان، كه جز علل شايع درد زانو در دختران محسوب مي‌شد، در دختران بيشتر از پسران ديده شد. بيماري اسگود- شلاتر و استئوكندريت ديسكانت در پسرها شايع‌تر بود. عللي همچون آرتريت روماتویید جوانان و استئوكندريت ديسكانت در مطالعات قبلي جز علل نادر درد مزمن جلوي زانو در نوجوانان گزارش شده بود، ولي در اين مطالعه از شيوع بيشتري برخوردار بود. در نهايت مي‌توان با دانش بيشتر در مورد علل درد مزمن زانو، بسياري از اين علل را تشخيص داد و درمان كرد و راه‌هاي پيشگيري ازآن‌ها را به بيماران آموزش داد.

Title: بررسي تأثیر تمرين درماني بر بهبود تعادل، راه رفتن، كيفيت زندگي و افسردگي افراد سالمند مبتلا به سرگيجه ناشي از ضايعات محيطي سيستم وستيبولار

http://jrrs.mui.ac.ir/index.php/jrrs/article/view/43

<!-- /* Font Definitions */ @font-face {font-family:"Cambria Math"; panose-1:2 4 5 3 5 4 6 3 2 4; mso-font-charset:1; mso-generic-font-family:roman; mso-font-format:other; mso-font-pitch:variable; mso-font-signature:0 0 0 0 0 0;} @font-face {font-family:"B Zar"; panose-1:0 0 4 0 0 0 0 0 0 0; mso-font-charset:178; mso-generic-font-family:auto; mso-font-pitch:variable; mso-font-signature:8193 -2147483648 8 0 64 0;} @font-face {font-family:"B Mitra"; panose-1:0 0 4 0 0 0 0 0 0 0; mso-font-charset:178; mso-generic-font-family:auto; mso-font-pitch:variable; mso-font-signature:8193 -2147483648 8 0 64 0;} @font-face {font-family:"B Nazanin"; panose-1:0 0 4 0 0 0 0 0 0 0; mso-font-charset:178; mso-generic-font-family:auto; mso-font-pitch:variable; mso-font-signature:8193 -2147483648 8 0 64 0;} /* Style Definitions */ p.MsoNormal, li.MsoNormal, div.MsoNormal {mso-style-unhide:no; mso-style-qformat:yes; mso-style-parent:""; margin:0cm; margin-bottom:.0001pt; text-align:right; mso-pagination:widow-orphan; direction:rtl; unicode-bidi:embed; font-size:12.0pt; font-family:"Times New Roman","serif"; mso-fareast-font-family:"Times New Roman"; mso-bidi-language:AR-SA;} p.matn, li.matn, div.matn {mso-style-name:matn; mso-style-unhide:no; mso-style-link:"matn Char"; margin:0cm; margin-bottom:.0001pt; text-align:justify; mso-pagination:widow-orphan; tab-stops:14.2pt; direction:rtl; unicode-bidi:embed; font-size:11.0pt; mso-bidi-font-size:13.0pt; font-family:"Times New Roman","serif"; mso-fareast-font-family:"Times New Roman"; mso-bidi-font-family:"B Mitra";} p.abst, li.abst, div.abst {mso-style-name:abst; mso-style-unhide:no; mso-style-link:"abst Char"; margin-top:3.0pt; margin-right:0cm; margin-bottom:0cm; margin-left:0cm; margin-bottom:.0001pt; text-align:justify; mso-pagination:widow-orphan; direction:rtl; unicode-bidi:embed; font-size:11.0pt; font-family:"Times New Roman","serif"; mso-fareast-font-family:"Times New Roman"; mso-bidi-font-family:"B Zar";} p.seri, li.seri, div.seri {mso-style-name:seri; mso-style-unhide:no; margin:0cm; margin-bottom:.0001pt; text-align:right; mso-pagination:widow-orphan; tab-stops:right 467.8pt; direction:rtl; unicode-bidi:embed; font-size:9.0pt; mso-bidi-font-size:10.0pt; font-family:"Times New Roman","serif"; mso-fareast-font-family:"Times New Roman"; mso-bidi-font-family:"B Nazanin"; mso-bidi-font-weight:bold;} span.matnChar {mso-style-name:"matn Char"; mso-style-unhide:no; mso-style-locked:yes; mso-style-link:matn; mso-ansi-font-size:11.0pt; mso-bidi-font-size:13.0pt; font-family:"B Mitra"; mso-bidi-font-family:"B Mitra";} span.abstChar {mso-style-name:"abst Char"; mso-style-unhide:no; mso-style-locked:yes; mso-style-link:abst; mso-ansi-font-size:11.0pt; mso-bidi-font-size:11.0pt; font-family:"B Zar"; mso-bidi-font-family:"B Zar";} .MsoChpDefault {mso-style-type:export-only; mso-default-props:yes; font-size:10.0pt; mso-ansi-font-size:10.0pt; mso-bidi-font-size:10.0pt;} @page Section1 {size:612.0pt 792.0pt; margin:72.0pt 72.0pt 72.0pt 72.0pt; mso-header-margin:36.0pt; mso-footer-margin:36.0pt; mso-paper-source:0;} div.Section1 {page:Section1;} --> مقدمه: سرگیجه یکی از علایم درگیری سیستم وستیبولار می‌باشد که با ایجاد حس چرخش در خود فرد یا محیط، بر هم خوردن تعادل بدن در حین انجام فعالیت‌های عملکردی، راه رفتن بدون کنترل و ایجاد افسردگی در افراد سالمند تظاهر می‌یابد. در اين مطالعه از تمرینات Cawthorne-cooksey به منظور بر طرف کردن علایم درگیری سیستم وستیبولار افراد سالمند مبتلا به ضایعات محیطی وستیبولار استفاده شد. مواد و روش‌ها: این مطالعه از نوع آینده‌نگر، شبه تجربی و غیر تصادفی بود و در سال 1386 در كلينيك فيزيوتراپي بیمارستان الزهرا (س) اصفهان انجام گرفت. از ميان بيماران سالمند مبتلا به سرگيجه ناشي از ضايعات محيطي وستيبولار مراجعه كننده به بيمارستان الزهرا (س)، 41 بیمار با داشتن شرایط ویژه به صورت غیر تصادفی انتخاب گردیدند. تمرینات طی سه مرحله به بیماران آموزش داده و از آن‌ها خواسته می‌شد که هر گروه از تمرینات را 3 بار در روز و هر بار 10 دقیقه به مدت یک هفته انجام دهند. قبل و بعد از درمان و بعد از یک ماه پی‌گیری جهت بررسی بالانس، راه رفتن، کیفیت زندگی و افسردگی بیماران به ترتیب از آزمون‌های Berg balance scale، Dynamic gait index، Dizziness handicap inventory و پرسش‌نامه Beck استفاده شد. همچنین برای تجزیه و تحلیل داده‌ها نیز از نرم‌افزار SPSS، آزمون‌های t زوج شده و Wilcoxon استفاده گردید. يافته‌ها: در ارزیابی بالانس بين قبل و بعد از درمان، قبل از درمان و بعد از پی‌گیری (Follow up) و بعد از درمان و بعد از Follow up تفاوت معني‌داري حاصل گرديد (001/0 > P). در ارزیابی راه رفتن بين قبل و بعد از درمان، قبل از درمان و بعد از Follow up و بعد از درمان و بعد از Follow up تفاوت معني‌داري حاصل گرديد (001/0 > P). در ارزیابی كيفيت زندگي بين قبل و بعد از درمان، قبل از درمان و بعد از Follow up و بعد از درمان و بعد از Follow up تفاوت معني‌داري حاصل گرديد (001/0 > P). در ارزیابی افسردگي بين قبل و بعد از درمان، قبل از درمان و بعد از Follow up و بعد از درمان و بعد از Follow up تفاوت معني‌داري حاصل گرديد (001/0 > P). بحث: با توجه به نتايج حاصل از تحقيق مي‌توان چنين نتيجه گرفت كه استفاده از تمرينات Cawthorne-cooksey باعث بهبود تعادل، راه رفتن،كيفيت زندگي و افسردگي افراد سالمند حداقل براي يك ماه مي‌گردد. البته بايد توجه داشت كه تمرينات بايد به طور روزانه توسط بيمار دنبال شود تا نتيجه مطلوب در دراز مدت حاصل گردد.

Title: ساخت متن فارسی برای ارزیابی صوت بزرگسالان و تعیین پایایی و روایی آن در اندازه‌گیری بسامد پایه و شدت گفتار

http://jrrs.mui.ac.ir/index.php/jrrs/article/view/44

مقدمه: در ارزيابي اختلالات صوت کوشش می‌شود که تا حد ممکن نمونه‌هاي صوت، گوياي صداي واقعي فرد در گفتار روزمره باشد. پژوهشگران دريافته‌اند كه ويژگي‌هاي صوت در خواندن متن به گفتار روزمره نزديك‌تر است. ساخت متن در بسياري از كشورها از اوايل قرن بيستم آغاز گشته است. عدم وجود متن مناسب در زبان فارسي، انجام پژوهش‌هاي مورد نياز و ارایه مستند نتايج اقدامات باليني را با مشكل مواجه كرده است. متن مورد استفاده در زبان فارسي براي پژوهش‌هاي صوت، آزمون لب‌خواني سارا بود كه پژوهشگران استفاده از آن را براي ارزيابي صوت نامناسب دانسته‌اند. هدف از انجام اين پژوهش، ساخت متن مناسبي براي ارزيابي ويژگي‌هاي صوت بود كه براي اهداف پژوهشي و باليني كارآمد باشد. مواد و روش‌ها: با مطالعه ويژگي‌هاي متن‌هاي مورد استفاده در ساير زبان‌ها، با توجه به وجود محدوديت نرم‌افزار Dr. Speech در تحلیل طول زمانی محرک‌ها و در نظر گرفتن نتایج حاصل از استفاده آزمون لب‌خواني سارا در پژوهش‌های گذشته، متن مورد نياز ساخته شد. متن ساخته شده از نظر روايي محتوا بررسي شد و پايايي آن نیز با استفاده از شيوه بازآزمايي تعيين گردید. یافته‌ها: متن تهيه شده از نظر روايي محتوا در نظرخواهي از استادان مورد تأیید قرار گرفت. در بررسي پايايي مشخص شد كه اندازه‌گيري ويژگي‌هاي مورد بررسي از پايايي بالايي برخوردار هستند. بحث: در بررسي روايي متن نتيجه حاصل از نظرخواهي از استادان، حداكثر توافق ممكن را نشان داد. در بررسي پايايي نيز ميزان آلفاي كرونباخ در متغيرهاي مورد مطالعه بالاتر از 7/0 به دست آمد. با توجه به تأیید روايي و دارا بودن پايايي مي‌توان نتيجه گرفت كه اين متن برای بررسي ويژگي‌هاي صوت در پژوهش‌هاي صوت در نمونه‌هاي عادي قابل استفاده خواهد بود.

Title: بررسی ناهنجاری کنترل نوسانات وضعیتی در شرايط ديناميکی و ارتباط آن با انعطاف‌پذيری تنه در زنان مبتلا به کمردرد مزمن

http://jrrs.mui.ac.ir/index.php/jrrs/article/view/45

<!-- /* Font Definitions */ @font-face {font-family:"Cambria Math"; panose-1:2 4 5 3 5 4 6 3 2 4; mso-font-charset:1; mso-generic-font-family:roman; mso-font-format:other; mso-font-pitch:variable; mso-font-signature:0 0 0 0 0 0;} @font-face {font-family:"B Zar"; panose-1:0 0 4 0 0 0 0 0 0 0; mso-font-charset:178; mso-generic-font-family:auto; mso-font-pitch:variable; mso-font-signature:8193 -2147483648 8 0 64 0;} /* Style Definitions */ p.MsoNormal, li.MsoNormal, div.MsoNormal {mso-style-unhide:no; mso-style-qformat:yes; mso-style-parent:""; margin:0cm; margin-bottom:.0001pt; text-align:right; mso-pagination:widow-orphan; direction:rtl; unicode-bidi:embed; font-size:12.0pt; font-family:"Times New Roman","serif"; mso-fareast-font-family:"Times New Roman"; mso-bidi-language:AR-SA;} p.abst, li.abst, div.abst {mso-style-name:abst; mso-style-unhide:no; margin-top:3.0pt; margin-right:0cm; margin-bottom:0cm; margin-left:0cm; margin-bottom:.0001pt; text-align:justify; mso-pagination:widow-orphan; direction:rtl; unicode-bidi:embed; font-size:10.0pt; mso-bidi-font-size:11.0pt; font-family:"Times New Roman","serif"; mso-fareast-font-family:"Times New Roman"; mso-bidi-font-family:"B Zar";} .MsoChpDefault {mso-style-type:export-only; mso-default-props:yes; font-size:10.0pt; mso-ansi-font-size:10.0pt; mso-bidi-font-size:10.0pt;} @page Section1 {size:612.0pt 792.0pt; margin:72.0pt 72.0pt 72.0pt 72.0pt; mso-header-margin:36.0pt; mso-footer-margin:36.0pt; mso-paper-source:0;} div.Section1 {page:Section1;} --> مقدمه: در تحقيقات پیشین کمردرد، به طور جداگانه، افت عملکرد بیومکانیکی مفاصل تنه از جمله انعطاف پذیری و ضعف عملکرد تعادلی در شرایط ایستا مورد تأکید قرار گرفته‌ است. اما اغلب شروع کمردرد در فعاليت‌های ديناميکي رخ مي‌دهد. بنابراين سنجش عملکرد تعادلی در شرايط ديناميکي از اهميت اساسي برخوردار است. هدف از اين مطالعه، شناسايي ناهنجاري کنترل وضعیت ديناميکي و ارتباط آن با انعطاف پذيري تنه در بيماران کمردرد مزمن بود. مواد و روش‌ها: تعداد 24 زن مبتلا به كمردرد مزمن و 24 زن سالم (25 تا 45 ساله)، به طور داوطلبانه در اين تحقيق شركت كردند. متغيرهاي نوسانات وضعیت در شرايط ديناميک، انعطاف پذیری کمر و درد كمر به ترتيب با استفاده از دستگاه تعادل سنج ديناميكي BIODEX، آزمون شوبر و پرسش‌نامه درد كيوبك اندازه‌گيري شد. داده‌هاي به دست آمده با استفاده از تکنيک آناليز واريانس چند متغيره‌ي ويژه داده‌هاي تکراري و روش همبستگي پيرسون با نرم‌افزار SPSS مورد تجزیه و تحليل آماري قرار گرفت. ميزان خطاي نوع اول در تمام آزمون‌ها 05/0 > α در نظر گرفته شد. یافته‌ها: در بيماران مبتلا به کمردرد مزمن، ناپايداري وضعیت در شرايط ديناميکي بيشتر بود (0001/0 = P). اين نارسايي در شرايط چشم بسته با وضوح بيشتري مشاهده شد. کاهش انعطاف پذیری در بيماران معنی‌دار بود (001/0 = P). بين عامل انعطاف‌پذيري و کنترل وضعیت، همبستگي ديده نشد. بحث: ناپايداري وضعیت ديناميکي در بيماران کمردردی در آزمون‌هاي مختلف و تشديد اين ناپايداري در حالت چشم بسته، نشان دهنده ضعف عملکردي گيرنده‌هاي حسي- عمقي اندام تحتاني و تنه آن‌ها است. عدم همبستگي بين انعطاف‌پذيري کمر با پايداري وضعیت نشان داد که در مراحل تشخيص و توانبخشي، اين دو عامل بايستي به طور مستقل مورد توجه قرار گيرند. تأکيد بر تقويت پايداري وضعیت در برنامه توانبخشي بيماران کمردردی توصيه مي‌شود. تحقيقات بيشتري براي تعيين اوليه يا ثانويه بودن عامل ناپايداري وضعیت در بيماران کمردردی لازم است

Title: تأثيرات وزن، جنس و تعداد حاملگي‌ها بر میزان زاویه لوردوز کامل و سگمنتال کمري و كمردرد افراد

http://jrrs.mui.ac.ir/index.php/jrrs/article/view/46

مقدمه: ستون فقرات بخش بسیار مهمی از چارچوب اسکلتی بدن انسان است و نقش اصلی را در حفظ راستای بدن ایفا می‌کند. هدف پژوهش حاضر، بررسی تأثیرات وزن، جنس و تعداد حاملگی‌ها بر روی لوردوز کامل و سگمنتال کمری افراد بود. مواد و روش‌ها: برای این منظور 75 نفر (41 مرد و 34 زن) با دامنه سنی 62-18 سال بدون هيچ‌گونه سابقه جراحی انتخاب شدند. اندازه‌گیری لوردوز کامل و سگمنتال با گرفتن رادیوگرافی از نمای جانبی ستون مهره‌ها در حالت ایستاده و با استفاده از روش Cobb انجام شد. متغیرهایی مانند جنس، قد، وزن، سن، شاخص توده بدنی (BMI) و تعداد زایمان آزمودنی‌ها بر روی برگه اطلاعات آن‌ها ثبت شد. برای روش‌های آماری از آزمون‌های ANOVA، آزمون تعقیبی توکی (Tokey) و آزمون t استفاده گردید (05/0 =P ). یافته‌ها: لوردوز کمری کامل در مردان 50 درجه و در زنان 54 درجه است و لوردوز سگمنتال کمر 2L-1L (مردان 3، زنان 5)، 3L-2L (مردان 7، زنان 5/8)، 4L-3L (مردان 9، زنان 10)، 5L-4L (مردان 12، زنان 5/14) و 1S-5L (مردان 22، زنان 5/18) می‌باشد. همچنین اضافه وزن در زنان تأثیر بیشتری در لوردوز مهره‌های بالای کمری نسبت به افراد با وزن عادی گذاشته بود. بحث: اضافه وزن، جنس و زایمان مکرر بر روی لوردوز کامل و سگمنتال کمری افراد مؤثر است. همچنین نتایج تحقیق نشان داد که با افزایش تعداد حاملگی، لوردوز کمر افزایش می‌یابد.

Title: ارزیابی شیوع ناهنجاری کیفوز در دانش آموزان دختر و پسر

http://jrrs.mui.ac.ir/index.php/jrrs/article/view/47

<!-- @page { margin: 0.79in } P { margin-bottom: 0.08in } --> مقدمه: کيفوز يکي از ناهنجاري‌هايي است که در دوران بچگي و نوجواني گسترش مي‌يابد و اغلب از نظرها پنهان مي‌ماند. اين ناهنجاري بيشتر در حين قرارگيري در وضعيت ناصحيح مشخص مي‌گردد و ممکن است در نتيجه تغييرات جدي در ساختمان مهره‌ها باشد. هدف از اين تحقيق، بررسي ميزان شيوع ناهنجاري كيفوز در دانش‌آموزان سه مقطع دبستان، راهنمايي و دبيرستان در دو گروه دختر و پسر شهرستان بندرعباس بود. مواد و روش‌ها: تعداد 502 نفر از دانش‌آموزان در سه مقطع دبيرستان (100 نفر دختر، 100 نفر پسر)، راهنمايي (47 نفر دختر، 57 نفر پسر) و دبستان (99 نفر دختر، 99 نفر پسر) از 24 مدرسه به عنوان نمونه آماري انتخاب شدند. براي به دست آوردن انحناي ستون فقرات از خط‌كش منعطف (دقت 1/0 درجه) و در نهايت جهت به دست آوردن زاويه كيفوز از روش Cobb استفاده شد. پس از به دست آوردن زوايا، مقادیر استاندارد جهت طبقه‌بندي افراد در 4 طبقه پشت صاف، طبیعی، كيفوز و كيفوز شديد مورد استفاده قرار گرفت. همچنین از آزمون 2χ براي تعيين نسبت در بين گروه‌ها استفاده شد. یافته‌ها: در مجموع از 502 نفر نمونه، 16 نفر پشت صاف، 393 نفر طبیعی، 92 نفر داراي كيفوز و 1 نفر داراي كيفوز شديد بودند. از مجموع 256 نفر پسر حاضر در اين تحقيق، 5 نفر پشت صاف (2 درصد)، 196 نفر طبیعی (6/76 درصد) و 55 نفر داراي كيفوز (4/21 درصد) و از بين 246 نفر دختر، 11 نفر داراي پشت صاف (5/4 درصد)، 197 نفر طبیعی (80 درصد)، 37 نفر داراي كيفوز (15درصد) و 1 نفر (5/0 درصد) با كيفوز شديد داشتند كه پس از انجام آزمون 2χ ، تفاوت معني‌داري در بين دو گروه پسر و دختر داراي كيفوز مشاهده نشد (05/0P > ). تعداد پسران و دختران دبستاني داراي كيفوز به ترتيب 22 و 11 نفر بودند که از لحاظ آماري تفاوت معني‌داري در بين اين دو گروه مشاهده شد (05/0 P <). تفاوت موجود ميان پسران راهنمايي (11 نفر) در مقايسه با دختران راهنمايي داراي كيفوز (9 نفر)، از لحاظ آماري معني‌دار نبود (05/0P > ). شيوع ناهنجاري کيفوز در بين پسران (22 نفر) و دختران دبيرستاني (17 نفر) از لحاظ آماري تفاوت معني‌داري نبود (05/0P > ). بحث: کاهش عادات غلط، انجام ورزش منظم و استفاده از پوشاك مناسب مي‌تواند از راه‌هاي کاهـــش ناهنجاري‌ها در دانش‌آموزان باشد.
